# (*E*)-6-Bromo-3-{2-[2-(2-chloro­benzyl­idene)hydrazin­yl]thia­zol-5-yl}-2*H*-chromen-2-one dimethyl sulfoxide monosolvate

**DOI:** 10.1107/S1600536811011160

**Published:** 2011-03-31

**Authors:** Afsheen Arshad, Hasnah Osman, Chan Kit Lam, Madhukar Hemamalini, Hoong-Kun Fun

**Affiliations:** a School of Chemical Sciences, Universiti Sains Malaysia, 11800 USM, Penang, Malaysia; b School of Pharmaceutical Sciences, Universiti Sains Malaysia, 11800 USM, Penang, Malaysia; c X-ray Crystallography Unit, School of Physics, Universiti Sains Malaysia, 11800 USM, Penang, Malaysia

## Abstract

In the title compound C_19_H_11_N_3_O_2_SClBr·C_2_H_6_OS, the mol­ecule adopts an *E* configuration about the central C=N double bond. The chromene ring system and the thia­zole ring are approximately planar, with maximum deviations of 0.027 (2) and 0.003 (1) Å, respectively. The central thia­zole ring makes dihedral angles of 21.82 (9) and 5.88 (7)° with the chloro-substituted phenyl ring and the chromene ring, respectively. In the crystal, mol­ecules are connected *via* N—H⋯O, N—H⋯S and C—H⋯O hydrogen bonds, forming supra­molecular chains along the *c* axis. An intra­molecular C—H⋯O hydrogen bond occurs. π–π inter­actions are observed between the thia­zole and phenyl rings [centroid–centroid distance = 3.6293 (10) Å]. A short Br⋯Cl contact of 3.37 (6) Å also occurs.

## Related literature

For details and applications of coumarin derivatives, see Liebig *et al.* (1974 ▶); Pathak *et al.* (1981 ▶); Hwu *et al.* (2008 ▶); Lee *et al.* (2003 ▶); Siddiqui *et al.* (2009 ▶). For the synthesis of the title compound, see: Tian *et al.* (1997 ▶); Yaragatti *et al.* (2010 ▶). For the stability of the temperature controller used in the data collection, see: Cosier & Glazer (1986 ▶).
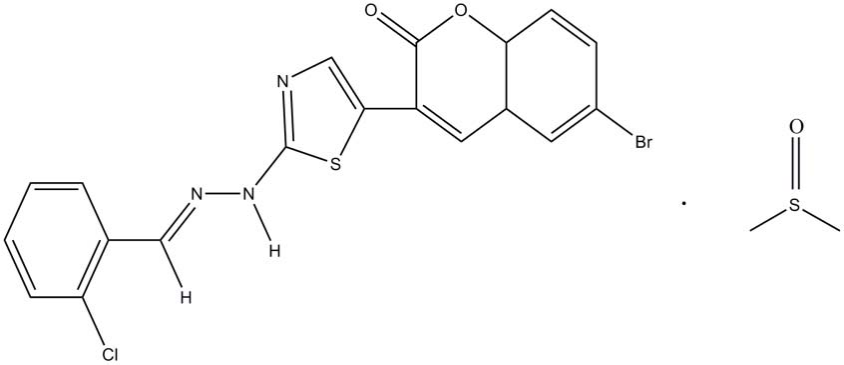


## Experimental

### 

#### Crystal data

C_19_H_11_BrClN_3_O_2_S·C_2_H_6_OS
*M* *_r_* = 538.86
Monoclinic, 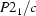

*a* = 6.5806 (4) Å
*b* = 15.7789 (9) Å
*c* = 20.9378 (13) Å
β = 90.684 (2)°
*V* = 2173.9 (2) Å^3^
*Z* = 4
Mo *K*α radiation
μ = 2.24 mm^−1^
*T* = 100 K
0.49 × 0.09 × 0.06 mm

#### Data collection

Bruker APEXII DUO CCD area-detector diffractometer
Absorption correction: multi-scan (*SADABS*; Bruker, 2009 ▶) *T*_min_ = 0.406, *T*_max_ = 0.870
37791 measured reflections
6392 independent reflections
5013 reflections with *I* > 2σ(*I*)
*R*_int_ = 0.059

#### Refinement

*R*[*F* ^2^ > 2σ(*F* ^2^)] = 0.032
*wR*(*F* ^2^) = 0.069
*S* = 1.00
6392 reflections
290 parameters
H atoms treated by a mixture of independent and constrained refinement
Δρ_max_ = 0.59 e Å^−3^
Δρ_min_ = −0.86 e Å^−3^

### 

Data collection: *APEX2* (Bruker, 2009 ▶); cell refinement: *SAINT* (Bruker, 2009 ▶); data reduction: *SAINT*; program(s) used to solve structure: *SHELXTL* (Sheldrick, 2008 ▶); program(s) used to refine structure: *SHELXTL*; molecular graphics: *SHELXTL*; software used to prepare material for publication: *SHELXTL* and *PLATON* (Spek, 2009 ▶).

## Supplementary Material

Crystal structure: contains datablocks global, I. DOI: 10.1107/S1600536811011160/sj5124sup1.cif

Structure factors: contains datablocks I. DOI: 10.1107/S1600536811011160/sj5124Isup2.hkl

Additional supplementary materials:  crystallographic information; 3D view; checkCIF report

## Figures and Tables

**Table 1:** Hydrogen-bond geometry (Å, °)

*D*—H⋯*A*	*D*—H	H⋯*A*	*D*⋯*A*	*D*—H⋯*A*
N2—H1*N*1⋯S2	0.85 (2)	2.81 (2)	3.5932 (17)	153 (2)
N2—H1*N*1⋯O3	0.85 (2)	1.97 (2)	2.808 (2)	169 (3)
C11—H11⋯O2	0.92 (2)	2.34 (3)	2.869 (2)	116.3 (19)
C13—H13*A*⋯O3	0.93	2.55	3.318 (2)	140
C17—H17*A*⋯O3^i^	0.93	2.60	3.285 (2)	131
C20—H20*C*⋯O2^ii^	0.96	2.47	3.431 (2)	176

Symmetry codes: (i) 
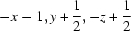
; (ii) 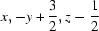.

**Fig. 1.**
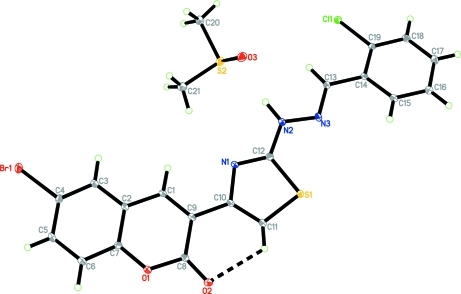
The asymmetric unit of the title compound, showing 30% probability displacement ellipsoids and the atom-numbering scheme. The intramolecular hydrogen bond is shown as a dashed line.

**Fig. 2.**
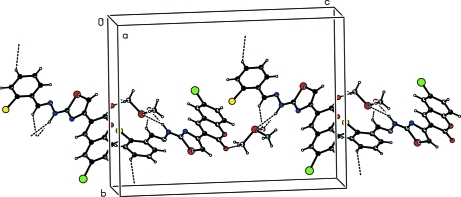
The crystal packing of the title compound (I).

## supplementary crystallographic information

### Comment

Coumarin derivatives have remarkable medicinal value due to their potential chemotherapeutic (Liebig *et al.*, 1974), fungicidal (Pathak *et al.*, 1981), antiviral (Hwu *et al.*, 2008) and anticoagulant (Lee *et al.*, 2003) properties. Furthermore, coumarins with a variety of substituted thiazole rings exhibit promising biological activities. Recently, some coumarins incorporating thiazolyl semicarbazones which act as anticonvulsant agents were reported (Siddiqui *et al.*, 2009). The title compound (I) is a new derivative of hydrazinyl thiazolyl coumarin. We present here its crystal structure.

The asymmetric unit of the title compound (Fig. 1) consists of one (*E*)-6-bromo-3-(2-(2-(2-chlorobenzylidene)hydrazinyl)thiazol-5-yl)- 2*H*-chromen-2-one molecule and one dimethylsulfoxide solvent molecule. The chromene (O1/C1–C9) ring system and thiazole (S1/N1/C10–C12) ring are approximately planar, with maximum deviations of 0.027 (2) Å for atom C9 and 0.003 (1)Å for atom N1, respectively.  The molecule adopts an *E* configuration about the central C13═N3 double bond.  The central thiazole (S1/N1/C10–C12) ring makes dihedral angles of 21.82 (9)° and 5.88 (7)° with the chloro-substituted phenyl (C14–C19) ring and the chromene (O1/C1–C9) ring, respectively.

In the crystal structure, (Fig. 2), the molecules are connected via N2—H1N1···S2, N2—H1N1···O3, C13—H13A···O3, C17—H17A···O3 and C20—H20C···O2 (Table 1) hydrogen bonds to form one dimensional supramolecular chains along the *c*-axis.  An intramolecular C11—H11···O2 hydrogen bond stabilizes the molecular structure. π···π interactions are observed between the thiazole (S1/N1/C10–C12) and phenyl (C2–C7) rings [centroid-centroid distance = 3.6293 (10)) Å; -1+x, y, z].  A short Br···Cl contact of 3.37 Å also occurs.

### Experimental

2-chlorobenzylidene thiosemicarbazone (Tian *et al.*, 1997) and 6-bromo-3-(2-bromoacetyl)-2*H*-chromen-2-one (Yaragatti *et al.*, 2010) were synthesized as reported in the literature. Title compound (I) was prepared by reacting 2-chlorobenzylidene thiosemicarbazone (2.5 mmol) with 6-bromo-3-(2-bromoacetyl)-2*H*- chromen-2-one (2.5 mmol) in chloroform-ethanol (3:1) mixture. The reaction mixture was refluxed for 2–3 hours at 60°C to yield a dense yellow precipitate. The mixture was cooled in ice bath and basified with ammonia to pH 7–8. The title compound (I) was recrystallized from DMSO as yellow needle-like crystals.

### Refinement

Atoms H11 and H1N1  were located from a difference Fourier map and refined freely [N–H = 0.95 (3) Å]. The remaining H atoms were positioned geometrically [C–H = 0.93 or 0.96 Å] and were refined using a riding model, with *U*_iso_(H) = 1.2 or 1.5 *U*_eq_(C).  A rotating group model was applied to the methyl groups.  The highest residual electron density peak is located at 0.78 Å from Br1 and the deepest hole is located at 0.68 Å from Br1.

### Crystal data

**Table:**

C_19_H_11_BrClN_3_O_2_S·C_2_H_6_OS	*F*(000) = 1088
*M**_r_* = 538.86	*D*_x_ = 1.646 Mg m^−^^3^
Monoclinic, *P*2_1_/*c*	Mo *K*α radiation, λ = 0.71073 Å
Hall symbol: -P 2ybc	Cell parameters from 9665 reflections
*a* = 6.5806 (4) Å	θ = 2.8–29.9°
*b* = 15.7789 (9) Å	µ = 2.24 mm^−^^1^
*c* = 20.9378 (13) Å	*T* = 100 K
β = 90.684 (2)°	Needle, yellow
*V* = 2173.9 (2) Å^3^	0.49 × 0.09 × 0.06 mm
*Z* = 4	

### Data collection

**Table:**

Bruker APEXII DUO CCD area-detector diffractometer	6392 independent reflections
Radiation source: fine-focus sealed tube	5013 reflections with *I* > 2σ(*I*)
graphite	*R*_int_ = 0.059
φ and ω scans	θ_max_ = 30.1°, θ_min_ = 1.6°
Absorption correction: multi-scan (*SADABS*; Bruker, 2009)	*h* = −9→9
*T*_min_ = 0.406, *T*_max_ = 0.870	*k* = −22→22
37791 measured reflections	*l* = −29→28

### Refinement

**Table:**

Refinement on *F*^2^	Primary atom site location: structure-invariant direct methods
Least-squares matrix: full	Secondary atom site location: difference Fourier map
*R*[*F*^2^ > 2σ(*F*^2^)] = 0.032	Hydrogen site location: inferred from neighbouring sites
*wR*(*F*^2^) = 0.069	H atoms treated by a mixture of independent and constrained refinement
*S* = 1.00	*w* = 1/[σ^2^(*F*_o_^2^) + (0.0237*P*)^2^ + 1.8103*P*] where *P* = (*F*_o_^2^ + 2*F*_c_^2^)/3
6392 reflections	(Δ/σ)_max_ = 0.002
290 parameters	Δρ_max_ = 0.59 e Å^−^^3^
0 restraints	Δρ_min_ = −0.86 e Å^−^^3^

### Special details

**Table:**

Experimental. The crystal was placed in the cold stream of an Oxford Cryosystems Cobra open-flow nitrogen cryostat (Cosier & Glazer, 1986) operating at 100.0 (1) K.
Geometry. All s.u.'s (except the s.u. in the dihedral angle between two l.s. planes) are estimated using the full covariance matrix. The cell s.u.'s are taken into account individually in the estimation of s.u.'s in distances, angles and torsion angles; correlations between s.u.'s in cell parameters are only used when they are defined by crystal symmetry. An approximate (isotropic) treatment of cell s.u.'s is used for estimating s.u.'s involving l.s. planes.
Refinement. Refinement of F^2^ against ALL reflections. The weighted R-factor wR and goodness of fit S are based on F^2^, conventional R-factors R are based on F, with F set to zero for negative F^2^. The threshold expression of F^2^ > 2σ(F^2^) is used only for calculating R-factors(gt) etc. and is not relevant to the choice of reflections for refinement. R-factors based on F^2^ are statistically about twice as large as those based on F, and R- factors based on ALL data will be even larger.

### Fractional atomic coordinates and isotropic or equivalent isotropic displacement parameters (Å^2^)

**Table:**

	*x*	*y*	*z*	*U*_iso_*/*U*_eq_	
Br1	1.24046 (3)	0.503519 (12)	0.413433 (9)	0.02077 (6)	
S1	0.06464 (7)	0.92776 (3)	0.44296 (2)	0.01447 (9)	
Cl1	−0.43250 (7)	0.82410 (3)	0.14834 (2)	0.01568 (9)	
S2	0.19587 (8)	0.69303 (3)	0.22076 (2)	0.01861 (10)	
O1	0.85351 (19)	0.78959 (8)	0.55746 (6)	0.0141 (3)	
O2	0.6219 (2)	0.88767 (8)	0.57238 (6)	0.0178 (3)	
O3	0.0131 (2)	0.67996 (9)	0.26280 (7)	0.0221 (3)	
N1	0.2804 (2)	0.79823 (9)	0.40883 (7)	0.0124 (3)	
N2	−0.0151 (2)	0.81708 (10)	0.34770 (8)	0.0151 (3)	
N3	−0.1775 (2)	0.86848 (9)	0.33469 (7)	0.0140 (3)	
C1	0.6516 (3)	0.73061 (11)	0.44975 (8)	0.0134 (3)	
H1A	0.5825	0.7093	0.4142	0.016*	
C2	0.8397 (3)	0.69208 (11)	0.46917 (8)	0.0131 (3)	
C3	0.9315 (3)	0.62551 (11)	0.43607 (9)	0.0151 (4)	
H3A	0.8693	0.6027	0.3998	0.018*	
C4	1.1151 (3)	0.59407 (11)	0.45784 (9)	0.0154 (4)	
C5	1.2114 (3)	0.62663 (12)	0.51219 (9)	0.0160 (4)	
H5A	1.3353	0.6045	0.5260	0.019*	
C6	1.1216 (3)	0.69197 (11)	0.54534 (9)	0.0155 (4)	
H6A	1.1836	0.7141	0.5818	0.019*	
C7	0.9373 (3)	0.72427 (11)	0.52342 (8)	0.0130 (3)	
C8	0.6760 (3)	0.82992 (11)	0.53893 (8)	0.0126 (3)	
C9	0.5713 (3)	0.79729 (11)	0.48163 (8)	0.0127 (3)	
C10	0.3801 (3)	0.83652 (11)	0.46033 (8)	0.0123 (3)	
C11	0.2874 (3)	0.90653 (11)	0.48441 (9)	0.0146 (3)	
C12	0.1142 (3)	0.83956 (11)	0.39565 (8)	0.0129 (3)	
C13	−0.2802 (3)	0.84836 (11)	0.28434 (8)	0.0135 (3)	
H13A	−0.2395	0.8028	0.2594	0.016*	
C14	−0.4611 (3)	0.89708 (10)	0.26614 (8)	0.0119 (3)	
C15	−0.5571 (3)	0.95147 (11)	0.30911 (8)	0.0140 (3)	
H15A	−0.5063	0.9561	0.3506	0.017*	
C16	−0.7256 (3)	0.99838 (11)	0.29128 (9)	0.0165 (4)	
H16A	−0.7852	1.0351	0.3204	0.020*	
C17	−0.8065 (3)	0.99079 (11)	0.22969 (9)	0.0174 (4)	
H17A	−0.9203	1.0222	0.2177	0.021*	
C18	−0.7171 (3)	0.93639 (11)	0.18634 (9)	0.0159 (4)	
H18A	−0.7713	0.9305	0.1454	0.019*	
C19	−0.5458 (3)	0.89069 (10)	0.20465 (8)	0.0124 (3)	
C20	0.1935 (3)	0.60863 (12)	0.16416 (9)	0.0197 (4)	
H20A	0.0794	0.6153	0.1356	0.030*	
H20B	0.1828	0.5555	0.1862	0.030*	
H20C	0.3170	0.6097	0.1402	0.030*	
C21	0.4090 (3)	0.66012 (13)	0.26810 (10)	0.0248 (4)	
H21A	0.4260	0.6980	0.3036	0.037*	
H21B	0.5291	0.6610	0.2426	0.037*	
H21C	0.3864	0.6037	0.2836	0.037*	
H11	0.337 (4)	0.9398 (15)	0.5171 (11)	0.028 (6)*	
H1N1	0.009 (4)	0.7740 (15)	0.3243 (11)	0.025 (6)*	

### Atomic displacement parameters (Å^2^)

**Table:**

	*U*^11^	*U*^22^	*U*^33^	*U*^12^	*U*^13^	*U*^23^
Br1	0.01991 (10)	0.02102 (9)	0.02135 (10)	0.00864 (8)	−0.00087 (7)	−0.00287 (8)
S1	0.0134 (2)	0.01564 (19)	0.0143 (2)	0.00403 (17)	−0.00280 (17)	−0.00214 (16)
Cl1	0.0156 (2)	0.01753 (19)	0.01388 (19)	0.00135 (17)	−0.00127 (16)	−0.00306 (15)
S2	0.0189 (2)	0.01360 (19)	0.0234 (2)	−0.00134 (18)	0.00188 (19)	0.00019 (17)
O1	0.0126 (6)	0.0165 (6)	0.0132 (6)	0.0020 (5)	−0.0029 (5)	−0.0014 (5)
O2	0.0173 (7)	0.0198 (6)	0.0162 (6)	0.0021 (5)	−0.0036 (5)	−0.0055 (5)
O3	0.0189 (7)	0.0204 (7)	0.0271 (8)	−0.0024 (6)	0.0065 (6)	−0.0067 (6)
N1	0.0112 (7)	0.0138 (7)	0.0121 (7)	0.0003 (6)	−0.0023 (6)	−0.0013 (5)
N2	0.0135 (7)	0.0161 (7)	0.0157 (7)	0.0036 (6)	−0.0049 (6)	−0.0032 (6)
N3	0.0109 (7)	0.0161 (7)	0.0149 (7)	0.0021 (6)	−0.0014 (6)	0.0019 (6)
C1	0.0133 (8)	0.0157 (8)	0.0111 (8)	−0.0001 (7)	−0.0035 (7)	−0.0002 (6)
C2	0.0110 (8)	0.0149 (8)	0.0134 (8)	−0.0003 (7)	−0.0014 (7)	0.0007 (6)
C3	0.0139 (9)	0.0170 (8)	0.0145 (8)	0.0017 (7)	−0.0028 (7)	−0.0008 (7)
C4	0.0153 (9)	0.0145 (8)	0.0164 (9)	0.0031 (7)	0.0011 (7)	−0.0002 (7)
C5	0.0125 (8)	0.0189 (8)	0.0166 (9)	0.0025 (7)	−0.0028 (7)	0.0038 (7)
C6	0.0146 (9)	0.0183 (8)	0.0136 (8)	−0.0008 (7)	−0.0031 (7)	0.0017 (7)
C7	0.0126 (8)	0.0135 (8)	0.0128 (8)	0.0000 (7)	−0.0004 (7)	−0.0004 (6)
C8	0.0104 (8)	0.0151 (8)	0.0123 (8)	−0.0010 (7)	−0.0004 (6)	0.0014 (6)
C9	0.0110 (8)	0.0150 (8)	0.0120 (8)	−0.0016 (7)	−0.0019 (7)	0.0016 (6)
C10	0.0103 (8)	0.0147 (8)	0.0117 (8)	0.0000 (7)	−0.0017 (6)	0.0010 (6)
C11	0.0128 (8)	0.0162 (8)	0.0147 (8)	0.0009 (7)	−0.0038 (7)	−0.0002 (7)
C12	0.0136 (8)	0.0125 (7)	0.0125 (8)	−0.0001 (7)	0.0002 (7)	0.0005 (6)
C13	0.0123 (8)	0.0135 (8)	0.0146 (8)	0.0010 (7)	−0.0017 (7)	−0.0004 (6)
C14	0.0103 (8)	0.0125 (7)	0.0129 (8)	−0.0014 (6)	−0.0013 (6)	0.0010 (6)
C15	0.0123 (8)	0.0168 (8)	0.0128 (8)	−0.0018 (7)	−0.0015 (7)	0.0013 (7)
C16	0.0159 (9)	0.0165 (8)	0.0172 (8)	0.0010 (7)	−0.0001 (7)	−0.0018 (7)
C17	0.0151 (9)	0.0181 (8)	0.0189 (9)	0.0032 (7)	−0.0022 (7)	0.0015 (7)
C18	0.0154 (9)	0.0178 (8)	0.0144 (8)	0.0004 (7)	−0.0043 (7)	0.0006 (7)
C19	0.0123 (8)	0.0115 (7)	0.0134 (8)	−0.0011 (6)	−0.0002 (7)	−0.0009 (6)
C20	0.0185 (9)	0.0228 (9)	0.0179 (9)	0.0006 (8)	0.0018 (8)	−0.0019 (7)
C21	0.0217 (10)	0.0269 (10)	0.0257 (10)	−0.0002 (9)	−0.0036 (8)	−0.0055 (8)

### Geometric parameters (Å, °)

**Table:**

Br1—C4	1.8986 (18)	C5—H5A	0.9300
S1—C11	1.7273 (18)	C6—C7	1.389 (2)
S1—C12	1.7413 (18)	C6—H6A	0.9300
Cl1—C19	1.7524 (18)	C8—C9	1.469 (2)
S2—O3	1.5130 (15)	C9—C10	1.467 (2)
S2—C20	1.7826 (19)	C10—C11	1.362 (2)
S2—C21	1.785 (2)	C11—H11	0.92 (2)
O1—C7	1.372 (2)	C13—C14	1.464 (2)
O1—C8	1.382 (2)	C13—H13A	0.9300
O2—C8	1.206 (2)	C14—C15	1.399 (2)
N1—C12	1.300 (2)	C14—C19	1.400 (2)
N1—C10	1.393 (2)	C15—C16	1.381 (2)
N2—C12	1.356 (2)	C15—H15A	0.9300
N2—N3	1.366 (2)	C16—C17	1.394 (3)
N2—H1N1	0.85 (2)	C16—H16A	0.9300
N3—C13	1.286 (2)	C17—C18	1.385 (3)
C1—C9	1.356 (2)	C17—H17A	0.9300
C1—C2	1.434 (2)	C18—C19	1.388 (2)
C1—H1A	0.9300	C18—H18A	0.9300
C2—C7	1.394 (2)	C20—H20A	0.9600
C2—C3	1.399 (2)	C20—H20B	0.9600
C3—C4	1.379 (2)	C20—H20C	0.9600
C3—H3A	0.9300	C21—H21A	0.9600
C4—C5	1.394 (2)	C21—H21B	0.9600
C5—C6	1.380 (3)	C21—H21C	0.9600
			
C11—S1—C12	88.12 (9)	C10—C11—S1	110.64 (13)
O3—S2—C20	106.51 (9)	C10—C11—H11	125.6 (15)
O3—S2—C21	105.18 (10)	S1—C11—H11	123.7 (15)
C20—S2—C21	98.77 (9)	N1—C12—N2	123.02 (16)
C7—O1—C8	122.92 (13)	N1—C12—S1	116.29 (13)
C12—N1—C10	109.50 (15)	N2—C12—S1	120.68 (13)
C12—N2—N3	118.35 (15)	N3—C13—C14	120.14 (16)
C12—N2—H1N1	120.9 (16)	N3—C13—H13A	119.9
N3—N2—H1N1	120.6 (16)	C14—C13—H13A	119.9
C13—N3—N2	114.80 (15)	C15—C14—C19	117.24 (16)
C9—C1—C2	121.96 (16)	C15—C14—C13	121.77 (15)
C9—C1—H1A	119.0	C19—C14—C13	120.99 (16)
C2—C1—H1A	119.0	C16—C15—C14	121.46 (16)
C7—C2—C3	118.67 (16)	C16—C15—H15A	119.3
C7—C2—C1	117.67 (16)	C14—C15—H15A	119.3
C3—C2—C1	123.65 (16)	C15—C16—C17	120.03 (17)
C4—C3—C2	119.21 (16)	C15—C16—H16A	120.0
C4—C3—H3A	120.4	C17—C16—H16A	120.0
C2—C3—H3A	120.4	C18—C17—C16	119.91 (17)
C3—C4—C5	121.69 (17)	C18—C17—H17A	120.0
C3—C4—Br1	119.54 (14)	C16—C17—H17A	120.0
C5—C4—Br1	118.76 (14)	C17—C18—C19	119.39 (16)
C6—C5—C4	119.52 (17)	C17—C18—H18A	120.3
C6—C5—H5A	120.2	C19—C18—H18A	120.3
C4—C5—H5A	120.2	C18—C19—C14	121.95 (16)
C5—C6—C7	119.07 (16)	C18—C19—Cl1	118.43 (13)
C5—C6—H6A	120.5	C14—C19—Cl1	119.61 (13)
C7—C6—H6A	120.5	S2—C20—H20A	109.5
O1—C7—C6	117.31 (15)	S2—C20—H20B	109.5
O1—C7—C2	120.85 (15)	H20A—C20—H20B	109.5
C6—C7—C2	121.84 (17)	S2—C20—H20C	109.5
O2—C8—O1	116.07 (15)	H20A—C20—H20C	109.5
O2—C8—C9	126.89 (16)	H20B—C20—H20C	109.5
O1—C8—C9	117.03 (15)	S2—C21—H21A	109.5
C1—C9—C10	121.02 (15)	S2—C21—H21B	109.5
C1—C9—C8	119.48 (16)	H21A—C21—H21B	109.5
C10—C9—C8	119.50 (16)	S2—C21—H21C	109.5
C11—C10—N1	115.44 (15)	H21A—C21—H21C	109.5
C11—C10—C9	128.05 (16)	H21B—C21—H21C	109.5
N1—C10—C9	116.52 (15)		
			
C12—N2—N3—C13	172.62 (17)	C1—C9—C10—C11	−175.39 (19)
C9—C1—C2—C7	1.4 (3)	C8—C9—C10—C11	5.5 (3)
C9—C1—C2—C3	−177.32 (18)	C1—C9—C10—N1	4.1 (3)
C7—C2—C3—C4	−0.2 (3)	C8—C9—C10—N1	−174.95 (16)
C1—C2—C3—C4	178.50 (17)	N1—C10—C11—S1	0.4 (2)
C2—C3—C4—C5	0.3 (3)	C9—C10—C11—S1	179.94 (15)
C2—C3—C4—Br1	179.80 (14)	C12—S1—C11—C10	−0.06 (15)
C3—C4—C5—C6	0.0 (3)	C10—N1—C12—N2	−179.95 (17)
Br1—C4—C5—C6	−179.46 (14)	C10—N1—C12—S1	0.6 (2)
C4—C5—C6—C7	−0.4 (3)	N3—N2—C12—N1	−175.07 (16)
C8—O1—C7—C6	176.78 (16)	N3—N2—C12—S1	4.4 (2)
C8—O1—C7—C2	−3.3 (3)	C11—S1—C12—N1	−0.32 (15)
C5—C6—C7—O1	−179.58 (16)	C11—S1—C12—N2	−179.80 (16)
C5—C6—C7—C2	0.5 (3)	N2—N3—C13—C14	178.34 (15)
C3—C2—C7—O1	179.92 (16)	N3—C13—C14—C15	−16.2 (3)
C1—C2—C7—O1	1.1 (3)	N3—C13—C14—C19	164.06 (17)
C3—C2—C7—C6	−0.2 (3)	C19—C14—C15—C16	−1.4 (3)
C1—C2—C7—C6	−178.99 (17)	C13—C14—C15—C16	178.83 (17)
C7—O1—C8—O2	−178.27 (16)	C14—C15—C16—C17	1.4 (3)
C7—O1—C8—C9	2.8 (2)	C15—C16—C17—C18	−0.3 (3)
C2—C1—C9—C10	179.09 (16)	C16—C17—C18—C19	−0.8 (3)
C2—C1—C9—C8	−1.8 (3)	C17—C18—C19—C14	0.8 (3)
O2—C8—C9—C1	−179.01 (18)	C17—C18—C19—Cl1	−178.63 (14)
O1—C8—C9—C1	−0.3 (2)	C15—C14—C19—C18	0.3 (3)
O2—C8—C9—C10	0.1 (3)	C13—C14—C19—C18	−179.91 (17)
O1—C8—C9—C10	178.85 (15)	C15—C14—C19—Cl1	179.71 (13)
C12—N1—C10—C11	−0.6 (2)	C13—C14—C19—Cl1	−0.5 (2)
C12—N1—C10—C9	179.78 (16)		

### Hydrogen-bond geometry (Å, °)

**Table:**

*D*—H···*A*	*D*—H	H···*A*	*D*···*A*	*D*—H···*A*
N2—H1N1···S2	0.85 (2)	2.81 (2)	3.5932 (17)	153 (2)
N2—H1N1···O3	0.85 (2)	1.97 (2)	2.808 (2)	169 (3)
C11—H11···O2	0.92 (2)	2.34 (3)	2.869 (2)	116.3 (19)
C13—H13A···O3	0.93	2.55	3.318 (2)	140
C17—H17A···O3^i^	0.93	2.60	3.285 (2)	131
C20—H20C···O2^ii^	0.96	2.47	3.431 (2)	176

Symmetry codes: (i) −*x*−1, *y*+1/2, −*z*+1/2; (ii) *x*, −*y*+3/2, *z*−1/2.
